# Gap Junctions, Dendrites and Resonances: A Recipe for Tuning Network Dynamics

**DOI:** 10.1186/2190-8567-3-15

**Published:** 2013-08-14

**Authors:** Yulia Timofeeva, Stephen Coombes, Davide Michieletto

**Affiliations:** 1 Department of Computer Science and Centre for Complexity Science, University of Warwick, Coventry, CV4 7AL, UK; 2 School of Mathematical Sciences, University of Nottingham, Nottingham, NG7 2RD, UK; 3 Complexity Science Doctoral Training Centre, University of Warwick, Coventry, CV4 7AL, UK

**Keywords:** Dendrites, Gap junctions, Resonant membrane, Sum-over-trips, Network dynamics

## Abstract

Gap junctions, also referred to as electrical synapses, are expressed along the entire central nervous system and are important in mediating various brain rhythms in both normal and pathological states. These connections can form between the dendritic trees of individual cells. Many dendrites express membrane channels that confer on them a form of sub-threshold resonant dynamics. To obtain insight into the modulatory role of gap junctions in tuning networks of resonant dendritic trees, we generalise the “sum-over-trips” formalism for calculating the response function of a single branching dendrite to a gap junctionally coupled network. Each cell in the network is modelled by a soma connected to an arbitrary structure of dendrites with resonant membrane. The network is treated as a single extended tree structure with dendro-dendritic gap junction coupling. We present the generalised “sum-over-trips” rules for constructing the network response function in terms of a set of coefficients defined at special branching, somatic and gap-junctional nodes. Applying this framework to a two-cell network, we construct compact closed form solutions for the network response function in the Laplace (frequency) domain and study how a preferred frequency in each soma depends on the location and strength of the gap junction.

## 1 Introduction

It has been known since the end of the nineteenth century and mainly from the work of Ramón y Cajal [1] that neuronal cells have a distinctive structure, which is different to that of any other cell type. The most extended parts of many neurons are dendrites. Their complex branching formations receive and integrate thousands of inputs from other cells in a network, via both chemical and electrical synapses. The voltage-dependent properties of dendrites can be uncovered with the use of sharp micropipette electrodes and it has long been recognised that modelling is essential for the interpretation of intracellular recordings. In the late 1950s, the theoretical work of Wilfrid Rall on cable theory provided a significant insight into the role of dendrites in processing synaptic inputs (see the book of Segev et al. [2] for a historical perspective on Rall’s work). Recent experimental and theoretical studies at a single cell level reinforce the fact that dendritic morphology and membrane properties play an important role in dendritic integration and firing patterns [3-5]. Coupling neuronal cells in a network adds an extra level of complexity to the generation of dynamic patterns. Electrical synapses, also known as gap junctions, are known to be important in mediating various brain rhythms in both normal [6,7] and pathological [8-10] states. They are mechanical and electrically conductive links between adjacent nerve cells that are formed at fine gaps between the pre- and post-synaptic cells and permit direct electrical connections between them. Each gap junction contains numerous connexon hemi-channels, which cross the membranes of both cells. With a lumen diameter of about 1.2 to 2.0 nm, the pore of a gap junction channel is wide enough to allow ions and even medium-sized signalling molecules to flow from one cell to the next thereby connecting the two cells’ cytoplasm. Being first discovered at the giant motor synapses of the crayfish in the late 1950s, gap junctions are now known to be expressed in the majority of cell types in the brain [11]. Without the need for receptors to recognise chemical messengers, gap junctions are much faster than chemical synapses at relaying signals. 

Earlier theoretical studies demonstrate that although neuronal gap junctions are able to synchronise network dynamics, they can also contribute toward the generation of many other dynamic patterns including anti-phase, phase-locked and bistable rhythms [12]. However, such studies often ignore dendritic morphology and focus only on somato-somatic gap junctions. In the case of dendro-dendritic coupling, simulations of multi-compartmental models reveal that network dynamics can be tuned by the location of the gap junction on the dendritic tree [13,14]. Here, we develop a more mathematical approach using the continuum cable description of a dendritic tree (either passive or resonant) that can compactly represent the response of an entire dendro-dendritic gap junction coupled neural network to injected current using a response function. This response function, often referred as a Green’s function, describes the voltage dynamics along a network structure in response to a delta-Dirac pulse applied at a given discrete location. Our work is based on the method for constructing the Green’s function of a single branched passive dendritic tree as originally proposed by Abbott et al. [15,16] and generalised by Coombes et al. [17] to treat resonant membrane (whereby subthreshold oscillatory behaviour is amplified for inputs at preferential frequencies determined by ionic currents such as $I_{h}$). This “sum-over-trips” method is built on the path integral formulation and calculates the Green’s function on an arbitrary dendritic geometry as a convergent infinite series solution.

In Sect. 2, we introduce the network model for gap junction coupled neurons. Each neuron in the network comprises of a soma and a dendritic tree. Cellular membrane dynamics are modelled by an ‘LRC’ (resonant) circuit. In Sect. 3, we focus on an example of two unbranched dendritic cells, with no distinguished somatic node, with identical and heterogeneous sets of parameters and give the closed form solution for network response with a single gap junction. The complete “sum-over-trips” rules for the more general case of an arbitrary network geometry are also presented. In Sect. 4, we apply the formalism to a more realistic case of two coupled neurons, each with a soma and a branching structure. We introduce a method of ‘words’ to construct compact solutions for the Green’s function of this network and study how a preferred frequency in each soma depends on the location and strength of the gap junction. Finally, in Sect. 5, we consider possible extensions of the work in this paper.

## 2 The Model

**Fig. 1:**
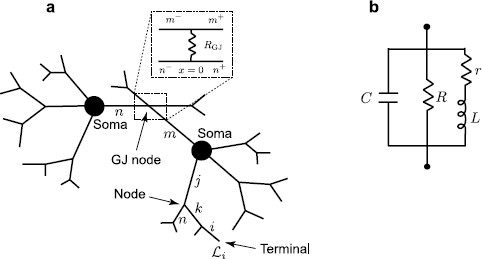
**a** A network of two cells connected by a gap junction (*GJ*). **b** An ‘LRC’ circuit modelling the resonant cell membrane

We consider a network of cells connected by gap junctions. The neural morphology of individual cells includes a branching dendritic structure and a lumped soma (see an illustrative example for two cells in Fig. 1a). We assume that the dendrites are not purely passive (i.e. modelled by the ‘RC’ circuit), but are resonant (i.e. modelled by the ‘LRC’ circuit shown in Fig. 1b). Many neurons exhibit resonances whereby subthreshold oscillatory behaviour is amplified for inputs at preferential frequencies, for example as seen in neurons of rat sensorimotor cortex [18]. In this case, it is known that the non-linear ionic current $I_{h}$ is responsible, and in general it is believed that the presence of $I_{h}$ in dendrites can have a major impact on the integration of subthreshold synaptic activity [19]. From a mathematical perspective, Mauro et al. [20] have shown that a linearisation of channel kinetics (for currents such as $I_{h}$), about rest, may adequately describe the observed resonant dynamics. The resulting linear system has a membrane impedance that displays resonant-like behaviour due to the additional presence of inductances (which are determined by the choice of channel model). This circuit is described by the specific membrane capacitance *C*, the resistance across a unit area of passive membrane *R* and an inductance *L* in series with a resistance *r*. The transmembrane voltage $V_{i}(x,t)$ on an individual branch *i* of each cell is then governed by the following set of equations:

(1) $$\frac{\partial V_{i}}{\partial t}=D_{i}\frac{\partial^{2}V_{i}}{\partial x^{2}}-\frac{V_{i}}{\tau_{i}}-\frac{1}{C_{i}}[I_{i}-I_{\mathrm{inj},i}],$$

(2) $$L_{i}\frac{\partial I_{i}}{\partial t}=-r_{i}I_{i}+V_{i},\;0\leq x\leq L_{i},t\geq 0.$$

The constants $D_{i}$ and $\tau_{i}$ can be found in terms of the electrical parameters of the cell membrane as $D_{i}=a_{i}/(4R_{a,i}C_{i})$ and $\tau_{i}=C_{i}R_{i}$, where $a_{i}$ is a diameter and $R_{a,i}$ is the specific cytoplasmic resistivity of branch *i*. The term $I_{\mathrm{inj},i}(x,t)$ models an external current applied to this branch. The dendritic structure of each cell is attached to an equipotential soma of the diameter $a_{s}$ modelled by the ‘LRC’ circuit with the parameters $C_{s}$, $R_{s}$, $L_{s}$ and $r_{s}$. Moreover, individual branches of different cells can be connected by gap junctions with a coupling parameter $R_{\mathrm{GJ}}$. 

Equations (1)–(2) for each dendritic segment must be accompanied with additional equations describing the dynamics of voltage at two ends of a segment. If the proximal (x=0) or distal ($x=L_{i}$) end of a branch is a branching node point the continuity of the potential across a node and Kirchoff’s law of conservation of current are imposed. For example, boundary conditions for a node indicated in Fig. 1a take the form:

(3) $$V_{j}(L_{j},t)=V_{n}(0,t)=V_{k}(0,t),$$

(4) $${\frac{1}{r_{a,j}}\frac{\partial V_{j}}{\partial x}|}_{x=L_{j}}={\frac{1}{r_{a,n}}\frac{\partial V_{n}}{\partial x}|}_{x=0}+{\frac{1}{r_{a,k}}\frac{\partial V_{k}}{\partial x}|}_{x=0},$$

where $r_{a,j}=4R_{a,j}/(\pi a_{j}^{2})$ is the axial resistance on branch *j*. If a branch terminates at $x=L_{i}$ we either have a no-flux (a closed-end) boundary condition

(5) $${\frac{\partial V_{i}}{\partial x}|}_{x=L_{i}}=0,$$

or a zero value (an open-end) boundary condition

(6) $$V_{i}(L_{i},t)=0.$$

A lumped soma can be treated as a special node point with the somatic membrane voltage $V_{s}(t)$ and the following set of equations, which imposes special boundary conditions on the proximal ends of branches connected to the soma:

(7) $$V_{s}(t)=V_{j}(0,t),$$

(8) $$C_{s}\frac{dV_{s}}{dt}=-\frac{V_{s}}{R_{s}}+{\sum_{j}\frac{1}{r_{a,j}}\frac{\partial V_{j}}{\partial x}|}_{x=0}-I_{s},$$

(9) $$L_{s}\frac{dI_{s}}{dt}=-r_{s}I_{s}+V_{s},$$

where the sum in Eq. (8) is over all branches connected to the soma. If the branches of two cells are coupled by a gap junction, the location of this coupling can be treated as a special node point on an extended branching structure. This gap-junctional (GJ) node requires the following set of boundary conditions (given here with an assumption that it is placed at x=0):

(10) $$V_{m^{-}}(0,t)=V_{m^{+}}(0,t),\;V_{n^{-}}(0,t)=V_{n^{+}}(0,t),$$

and

(11) $$\frac{1}{r_{a,m}}\left({\frac{\partial V_{m^{-}}}{\partial x}|}_{x=0}+{\frac{\partial V_{m^{+}}}{\partial x}|}_{x=0}\right)=g_{\mathrm{GJ}}\left(V_{m^{-}}(0,t)-V_{n^{-}}(0,t)\right),$$

(12) $$\frac{1}{r_{a,n}}\left({\frac{\partial V_{n^{-}}}{\partial x}|}_{x=0}+{\frac{\partial V_{n^{+}}}{\partial x}|}_{x=0}\right)=g_{\mathrm{GJ}}\left(V_{n^{-}}(0,t)-V_{m^{-}}(0,t)\right),$$

where $g_{\mathrm{GJ}}=1/R_{\mathrm{GJ}}$ is the conductance of the gap junction and $m^{-}$ and $m^{+}$ ($n^{-}$ and $n^{+}$) are two segments of branch *m* (branch *n*) connected at the gap junction (see Fig. 1a). The expressions in (10) reflect continuity of the potential across individual branches *m* and *n*, and Eqs. (11)–(12) enforce conservation of current.

A whole network model can be viewed as an extended tree structure with each individual node belonging to one of the following categories: a terminal, a regular branching node, a somatic node or the GJ node. The voltage dynamics along the network structure are described by linear equations and, therefore, the model’s behaviour can be studied by constructing the network response function known as the Green’s function, $G_{ij}(x,y,t)$. This function describes the voltage response at the location *x* on branch *i* in response to a delta-Dirac pulse applied to the location *y* on branch *j* at time t=0 (branches *i* and *j* can belong either to the same cell or to the two different cells). Knowing the Green’s function for the whole structure, it is easy to compute the voltage dynamics along the whole network for any form of an external input $I_{\mathrm{inj},j}(x,t)$ applied to branch *j* as

(13) $$\begin{array}{rcl} V_{i}(x,t) & = & \sum_{k}\int_{0}^{L_{k}}dyG_{ik}(x,y,t)V_{k}(y,0)\\ & & +\int_{0}^{t}ds\int_{0}^{L_{j}}dyG_{ij}(x,y,t-s)I_{\mathrm{inj},j}(y,s), \end{array}$$

where $V_{k}(x,0)$ describes the initial conditions on branch *k* and the sum is over all branches of the tree. Multiple external stimuli can be tackled by simply adding new terms with additional inputs $I_{\mathrm{inj},j}(x,t)$ to Eq. (13).

## 3 The Green’s Function on a Network

Earlier work of Coombes et al. [17] demonstrated that the Green’s function for a single cell with resonant membrane can be constructed by generalising the “sum-over-trips” framework of Abbott et al. [15,16] for passive dendrites. Here, we demonstrate how this framework can be extended to a network level starting with the simple case of two identical cells. 

### 3.1 Two Simplified Identical Cells

**Fig. 2:**
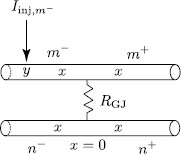
A network of two identical cells, each consists of an infinite dendritic cable, coupled by a gap junction

We consider the case of two identical cells coupled by a gap junction. Each cell consists of a single resonant dendrite of infinite length (see Fig. 2). A gap junction controlled by the parameter $R_{\mathrm{GJ}}$ and located at x=0 divides two dendrites into four semi-infinite segments: $m^{-}$, $m^{+}$, $n^{-}$, and $n^{+}$. We assume that an external input $I_{\mathrm{inj},m^{-}}(x,t)=\delta (x-y)\delta (t)$ is applied to segment $m^{-}$. The Green’s function on each segment must satisfy the set of Eqs. (1)–(2) with the boundary conditions at the gap junction given by Eqs. (10)–(12). Introducing the Laplace transform with spectral parameter *ω*

$$L\left[f(t)\right]=\hat{f}(\omega )=\int_{0}^{\infty }e^{-\omega t}f(t)\;dt,$$

and assuming zero initial data, we can solve this model in the frequency domain:

(14) $${\hat{G}}_{m^{-}}(x,y,\omega )=\frac{1}{C}\left[\frac{e^{-\gamma (\omega )|x-y|}}{2D\gamma (\omega )}-p_{\mathrm{GJ}}(\omega )\frac{e^{-\gamma (\omega )|x+y|}}{2D\gamma (\omega )}\right],$$

(15) $${\hat{G}}_{m^{+}}(x,y,\omega )=\frac{1}{C}\left[\left(1-p_{\mathrm{GJ}}(\omega )\right)\frac{e^{-\gamma (\omega )|x+y|}}{2D\gamma (\omega )}\right],$$

(16) $${\hat{G}}_{n^{-}}(x,y,\omega )={\hat{G}}_{n^{+}}(x,y,\omega )=\frac{1}{C}\left[p_{\mathrm{GJ}}(\omega )\frac{e^{-\gamma (\omega )|x+y|}}{2D\gamma (\omega )}\right],$$

where

(17) $$\gamma^{2}(\omega )=\frac{1}{D}\left[\frac{1}{\tau }+\omega +\frac{1}{C(r+\omega L)}\right],$$

and

(18) $$p_{\mathrm{GJ}}(\omega )=\frac{1}{2(z(\omega )R_{\mathrm{GJ}}+1)},\;z(\omega )=\gamma (\omega )/r_{a}.$$

**Fig. 3:**
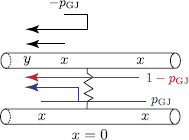
All trips (including the shortest trip for ${\hat{G}}_{m^{-}}(x,y,\omega )$ shown by *a dashed arrow*) and the corresponding coefficients $A_{\mathrm{trip}}(\omega )$ at the GJ for two identical cells

Solutions (14)–(16) are obtained using the “sum-over-trips” method where $\hat{G}(x,y,\omega )$ on each segment can be found as $\sum_{\mathrm{trips}}A_{\mathrm{trip}}(\omega ){\hat{G}}_{\infty }(L_{\mathrm{trip}},\omega )$, and

(19) $${\hat{G}}_{\infty }(x,\omega )=\frac{e^{-\gamma (\omega )|x|}}{2D\gamma (\omega )}$$

is the Laplace transform of the Green’s function $G_{\infty }(x,t)$ for an infinite resonant cable. $L_{\mathrm{trip}}$ is the length of a path that starts at point *x* on one of the segments and ends at point *y* on segment $m^{-}$. The trip coefficients $A_{\mathrm{trip}}(\omega )$ which ensure that the boundary conditions at the gap junction hold are chosen according to the following rules (see Fig. 3): 

• $A_{\mathrm{trip}}(\omega )=-p_{\mathrm{GJ}}(\omega )$ if the trip reflects along on the gap junction back onto the same dendrite.

• $A_{\mathrm{trip}}(\omega )=1-p_{\mathrm{GJ}}(\omega )$ if the trip passes through the gap junction along the same dendrite.

• $A_{\mathrm{trip}}(\omega )=p_{\mathrm{GJ}}(\omega )$ if the trip passes through the gap junction from one cell to another cell.

**Fig. 4:**
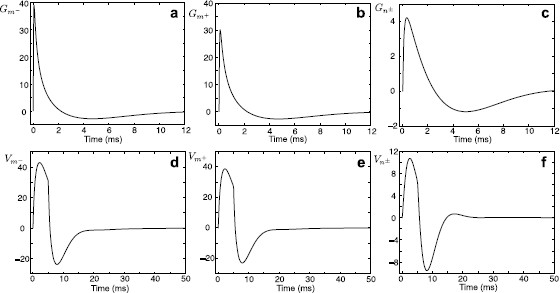
**a**–**c** The Green’s functions $G_{m^{-}}(x,y,t)$, $G_{m^{+}}(x,y,t)$, and $G_{n^{\pm }}(x,y,t)$ for a model in Fig. 2 when x=10 μm and y=100 μm. Parameters: a=2 μm, $D=50000{\text{ μm}}^{2}\text{/ms}$, τ=2 ms, $C=1{\text{ μF/cm}}^{2}$, $R_{a}=100\;\Omega \text{ cm}$, $r=100\;\Omega {\text{ cm}}^{2}$, $L=5{\text{ H cm}}^{2}$, $R_{\mathrm{GJ}}=100\text{ M}\Omega$. **d**–**f** Voltage profiles on each segment in response to a rectangular pulse of strength $\eta_{0}=2\text{ nA}$ and duration $\tau_{R}=5\text{ ms}$ applied to segment $m^{-}$. Note that different *y*-*axis* limits are used in **c** and **f**

Performing the numerical inverse Laplace transform ($L^{-1}$) of Eqs. (14)–(16), we obtain the Green’s function in the time domain for each segment. These Green’s functions are plotted in Figs. 4a–c. For any arbitrary form of external input $I_{\mathrm{inj},m^{-}}(x,t)=\delta (x-y)I(t)$, the voltage response on each segment can be found by taking a convolution of the corresponding Green’s function with this stimulus. Using the Laplace representation of the Green’s function on each segment given by Eqs. (14)–(16) this can be computed as

(20) $$V_{k}(x,t)=L^{-1}\left[{\hat{G}}_{k}(x,y,\omega )\hat{I}(\omega )\right],\;k\in \left\{m^{-},m^{+},n^{-},n^{+}\right\},$$

where $\hat{I}(\omega )=L[I(t)]$. In Figs. 4d–f, we plot the voltage profiles on each segment in response to a rectangular pulse $I(t)=\eta_{0}\Theta (t)\Theta (\tau_{R}-t)$ of strength $\eta_{0}$ and duration $\tau_{R}$, where Θ(t) is the Heaviside step function. 

**Fig. 5:**
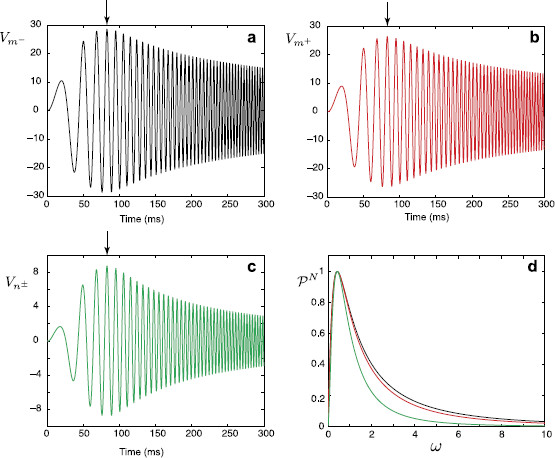
**a**–**c** Voltage profiles on each segment at x=10 μm in response to a stimulus $I_{\mathrm{chirp}}(t)$ applied at y=100 μm on segment $m^{-}$. Cells’ parameters as in Fig. 4, $\omega_{\mathrm{chirp}}=0.003$, $A_{\mathrm{chirp}}=1\text{ nA}$. **d** Normalised power functions $P_{m^{-}}^{N}(x,y,\omega )$ (*black curve*), $P_{m^{+}}^{N}(x,y,\omega )$ (*red curve*), $P_{n^{\pm }}^{N}(x,y,\omega )$ (*green curve*)

A response of the network model is characterised by the Green’s function and can be studied by introducing a power function $P_{k}(x,y,\omega )$ defined as $P_{k}(x,y,\omega )=|{\hat{G}}_{k}(x,y,\omega ){|}^{2}$. Resonant dynamics of the model for a given pair of locations (x,y) are directly linked with a value $\Omega_{0}$ at which the function $P_{k}(x,y,\omega )$ has its maximum. In Figs. 5a–c, we plot the voltage profiles on each segment in response to a chirp stimulus $I_{\mathrm{chirp}}(t)=A_{\mathrm{chirp}}\sin(\omega_{\mathrm{chirp}}t^{2})$. These figures clearly demonstrate resonant behaviour of the system maximising the voltage responses for particular frequencies. In Fig. 5d, we plot the normalised power functions $P_{k}^{N}(x,y,\omega )=P_{k}(x,y,\omega )/\max_{\omega }[P_{k}(x,y,\omega )]$ at the same locations. These power functions have their maximum at the value $\Omega_{0}=0.4271$, the same for each segment and, therefore, the resonances (indicated by arrows) in Figs. 5a–c occur at the same time. We can also notice that the function $P_{n^{\pm }}^{N}(x,y,\omega )$ decays to zero more rapidly than the functions $P_{m^{-}}^{N}(x,y,\omega )$ and $P_{m^{+}}^{N}(x,y,\omega )$. This explains the rapid reduction of voltage amplitude straight after the resonance in Fig. 5c. 

**Fig. 6:**
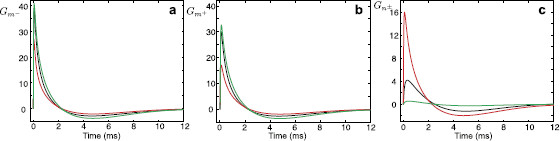
**a**–**c** The Green’s functions $G_{m^{-}}(x,y,t)$, $G_{m^{+}}(x,y,t)$, and $G_{n^{\pm }}(x,y,t)$ for a model in Fig. 2 when x=10 μm and y=100 μm. Parameters: a=2 μm, $D=50000{\text{ μm}}^{2}\text{/ms}$, τ=2 ms, $C=1{\text{ μF/cm}}^{2}$, $R_{a}=100\;\Omega \text{ cm}$, $r=100\;\Omega {\text{ cm}}^{2}$, $L=5{\text{ H cm}}^{2}$, $R_{\mathrm{GJ}}=100\text{ M}\Omega$ (*black curves*, as in Figs. 4a–c), $R_{\mathrm{GJ}}=1\text{ M}\Omega$ (*red curves*), $R_{\mathrm{GJ}}=1000\text{ M}\Omega$ (*green curves*). Note that different *y*-*axis* limits are used in **c**

Typical values of a unitary gap junction conductance are 10–550 pS [11] giving $g_{\mathrm{GJ}}=10\text{–}5500\text{ pS}$ (or $R_{\mathrm{GJ}}=180\text{–}10^{5}\text{ M}\Omega$) for 1–10 gap junction channels per electrical connection, although these estimates may be conservative and conductances from a larger range could be considered, as for example $g_{\mathrm{GJ}}=10\text{–}240000\text{ pS}$ corresponding to $R_{\mathrm{GJ}}=4\text{–}10^{5}\text{ M}\Omega$ in [14]. To demonstrate how the resistance of the gap junction affects the response function in a model of two identical cells, we plot Fig. 6 for $R_{\mathrm{GJ}}=100\text{ M}\Omega$ (black curves, the case shown in Figs. 4a–c), $R_{\mathrm{GJ}}=1$ MΩ (red curves) and $R_{\mathrm{GJ}}=1000\text{ M}\Omega$ (green curves). Low gap-junctional resistance significantly increases the amplitude of the Green’s function for Cell *n* and reduces the amplitude of the Green’s function for Cell *m*. Increasing the resistance reduces the response in Cell *n* and slightly increases the response in Cell *m*. In this model of two identical cells, the change of the resistance of the gap junction does not affect the resonant frequency $\Omega_{0}$, which is the same for each segment. 

**Fig. 7:**
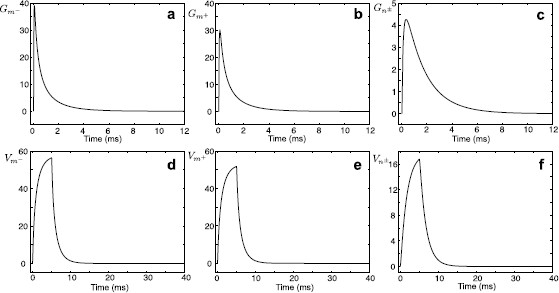
**a**–**c** The Green’s functions $G_{m^{-}}(x,y,t)$, $G_{m^{+}}(x,y,t)$, and $G_{n^{\pm }}(x,y,t)$ for a model in Fig. 2 with passive membrane when x=10 μm and y=100 μm. Parameters: a=2 μm, $D=50000{\text{ μm}}^{2}\text{/ms}$, τ=2 ms, $C=1{\text{ μF/cm}}^{2}$, $R_{a}=100\;\Omega \text{ cm}$, $R_{\mathrm{GJ}}=100\text{ M}\Omega$. **d**–**f** Voltage profiles on each segment in response to a rectangular pulse of strength $\eta_{0}=2\text{ nA}$ and duration $\tau_{R}=5\text{ ms}$ applied to segment $m^{-}$. Note that different *y*-*axis* limits are used in **c** and **f**

Although solutions (14)–(16) are found for the case of the resonant membrane, an ‘LRC’ circuit can be naturally turned into a ‘RC’ circuit by using the limit r→∞ which gives $\gamma^{2}(\omega )=(1/\tau +\omega )/D$. In the case of purely passive membrane, it is possible to make extra progress and find analytical forms of the solutions in the time domain (see Appendix A). In Figs. 7a–c, we plot the Green’s functions for the model in Fig. 2 with passive (instead of resonant) membrane. Voltage responses on each segment in response to a rectangular pulse are shown in Figs. 7d–f. 

### 3.2 Two Simplified Non-identical Cells

Here, we consider a model in Fig. 2 with the assumption that the cells are non-identical. Then using the Laplace transform and solving the model (with zero initial data) in the frequency domain, we obtain

(21) $${\hat{G}}_{m^{-}}(x,y,\omega )=\frac{1}{C_{m}}\left[\frac{e^{-\gamma_{m}(\omega )|x-y|}}{2D_{m}\gamma_{m}(\omega )}-p_{\mathrm{GJ},n}(\omega )\frac{e^{-\gamma_{m}(\omega )|x+y|}}{2D_{m}\gamma_{m}(\omega )}\right],$$

(22) $${\hat{G}}_{m^{+}}(x,y,\omega )=\frac{1}{C_{m}}\left[\left(1-p_{\mathrm{GJ},n}(\omega )\right)\frac{e^{-\gamma_{m}(\omega )|x+y|}}{2D_{m}\gamma_{m}(\omega )}\right],$$

(23) $${\hat{G}}_{n^{-}}(x,y,\omega )={\hat{G}}_{n^{+}}(x,y,\omega )=\frac{1}{C_{m}}\left[p_{\mathrm{GJ},m}(\omega )\frac{e^{-|\gamma_{n}(\omega )x+\gamma_{m}(\omega )y|}}{2D_{m}\gamma_{m}(\omega )}\right],$$

where the parameters $\gamma_{m}(\omega )$ and $\gamma_{n}(\omega )$ are defined in terms of cells’ individual properties as

(24) $$\gamma_{m}^{2}(\omega )=\frac{1}{D_{m}}\left[\frac{1}{\tau_{m}}+\omega +\frac{1}{C_{m}(r_{m}+\omega L_{m})}\right],$$

(25) $$\gamma_{n}^{2}(\omega )=\frac{1}{D_{n}}\left[\frac{1}{\tau_{n}}+\omega +\frac{1}{C_{n}(r_{n}+\omega L_{n})}\right].$$

**Fig. 8:**
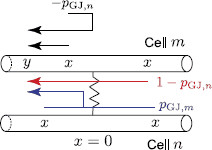
All trips (including the shortest trip for ${\hat{G}}_{m^{-}}(x,y,\omega )$ shown by *a dashed arrow*) and the corresponding coefficients $A_{\mathrm{trip}}(\omega )$ at the GJ for two non-identical cells

Solutions (21)–(23) show that the trip coefficients $A_{\mathrm{trip}}(\omega )$ depend on either $p_{\mathrm{GJ},m}(\omega )$ or $p_{\mathrm{GJ},n}(\omega )$ (see Fig. 8), which have the forms

(26) $$p_{\mathrm{GJ},m}(\omega )=\frac{z_{m}(\omega )}{z_{m}(\omega )+z_{n}(\omega )+2R_{\mathrm{GJ}}z_{m}(\omega )z_{n}(\omega )},\;z_{m}(\omega )=\gamma_{m}(\omega )/r_{a,m},$$

(27) $$p_{\mathrm{GJ},n}(\omega )=\frac{z_{n}(\omega )}{z_{m}(\omega )+z_{n}(\omega )+2R_{\mathrm{GJ}}z_{m}(\omega )z_{n}(\omega )},\;z_{n}(\omega )=\gamma_{n}(\omega )/r_{a,n}.$$

**Fig. 9:**
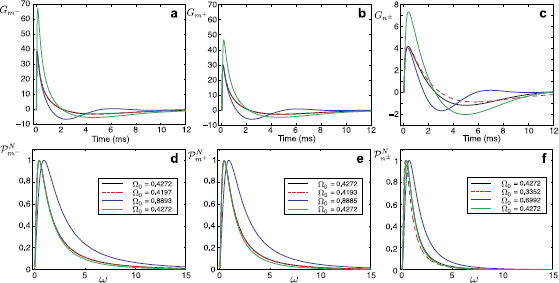
The Green’s functions $G_{m^{-}}(x,y,t)$, $G_{m^{+}}(x,y,t)$, and $G_{n^{\pm }}(x,y,t)$ (**a**–**c**) and normalised power functions $P_{m^{-}}^{N}(x,y,\omega )$, $P_{m^{+}}^{N}(x,y,\omega )$, and $P_{n^{\pm }}^{N}(x,y,\omega )$ (**d**–**f**) for a model in Fig. 2 when x=10 μm and y=100 μm. *Black curves*: two identical cells with the parameters as in Fig. 4. *Dashed red curves*: as in an identical case except $L_{n}=25{\text{ H cm}}^{2}$. *Blue curves*: as in an identical case except $L_{m}=1{\text{ H cm}}^{2}$. *Green curves*: as in an identical case except $a_{m}=1\text{ μm}$

In Figs. 9a–c, we demonstrate how individual variations in cell parameters affect the voltage response in the system. For each set of the parameters, we plot the Green’s functions $G_{m^{-}}(x,y,t)$, $G_{m^{+}}(x,y,t)$, and $G_{n^{\pm }}(x,y,t)$ obtained by taking the numerical inverse Laplace transform of (21)–(23). Black curves show the profiles for two identical cells. Dashed red curves are the Green’s functions for a case when $L_{n}$ is changed from $5\;{\text{H cm}}^{2}$ to $25\;{\text{H cm}}^{2}$. This change affects the response in Cell *n*, but not in Cell *m*. Blue curves are plotted for a case when $L_{m}$ is changed from $5\;{\text{H cm}}^{2}$ to $1\;{\text{H cm}}^{2}$. It has noticeable effect on both cells. Finally, green curves are plotted for the case $a_{m}=1\;\text{μm}$ instead of the original diameter $a_{m}=2\;\text{μm}$ showing changes in profiles in both cells. As the stimulus in these examples is applied to Cell *m*, any variations in the parameters of this cell have an immediate effect on the responses in Cell *n*. In contrast, Cell *m* seems to be mostly robust to variations in parameters in Cell *n*. Resonant properties of the cells’ responses can be studied by plotting the normalised power functions $P_{k}^{N}(x,y,\omega )$ for each of the parameter sets (see Figs. 9d–f). The heterogeneity of the cells’ parameters leads to appearances of different values of $\Omega_{0}$ (the maximum of the power function) for each cell. We can also notice that the power functions for Cell *n* are more localised around their peaks (Fig. 9f) in comparison to the power functions for Cell *m* (Figs. 9d, e) as it has been earlier observed in the case of two identical cells. 

### 3.3 An Arbitrary Network Geometry

**Fig. 10:**
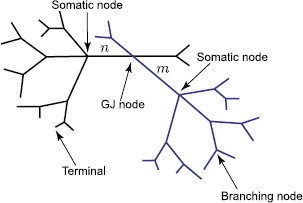
A network of two cells as an extended tree structure with different types of nodes

Here, we consider a network of spatially-extended cells of arbitrary geometries. This network can be treated as a single extended tree structure which consists of individual branches (indexed by a finite non-repeating sequence {1,2,…,i,…,k,…,j,…}) and three types of nodes: a regular branching node, a somatic node and a GJ node (see Fig. 10). The Green’s function $G_{ij}(x,y,t)$ for the whole structure can be found by obtaining ${\hat{G}}_{ij}(x,y,\omega )$ in the Laplace domain and then performing $L^{-1}[{\hat{G}}_{ij}(x,y,\omega )]$. We consider a general case when each branch of the network can have different biophysical parameters and is characterised by the function $\gamma_{k}(\omega )$ defined as

(28) $$\gamma_{k}^{2}(\omega )=\frac{1}{D_{k}}\left[\frac{1}{\tau_{k}}+\omega +\frac{1}{C_{k}(r_{k}+\omega L_{k})}\right],$$

where *k* labels an arbitrary branch of the network. Using the “sum-over-trips” formalism ${\hat{G}}_{ij}(x,y,\omega )$ can be constructed as an infinite series expansion

(29) $${\hat{G}}_{ij}(x,y,\omega )=\frac{1}{D_{j}\gamma_{j}(\omega )}\sum_{\mathrm{trips}}A_{\mathrm{trip}}(\omega ){\hat{H}}_{\infty }\left(L_{\mathrm{trip}}(i,j,x,y,\omega )\right),$$

where ${\hat{H}}_{\infty }(x)=e^{-|x|}/2$ and $L_{\mathrm{trip}}(i,j,x,y,\omega )$ is the length of a path along the network structure that starts at the point $\gamma_{i}(\omega )x$ on branch *i* and ends at the point $\gamma_{j}(\omega )y$ on branch *j*. Note that the length of each branch of the network needs to be scaled by $\gamma_{k}(\omega )$ before $L_{\mathrm{trip}}$ is calculated for (29). It is also worth mentioning here that if all branches of a network have the same biophysical parameters, i.e. $\gamma_{k}(\omega )=\gamma (\omega )$, the function ${\hat{H}}_{\infty }(L_{\mathrm{trip}}(\omega ))/(D\gamma (\omega ))={\hat{G}}_{\infty }(L_{\mathrm{trip}},\omega )$ defined by (19). The trip coefficients $A_{\mathrm{trip}}(\omega )$ in (29) are chosen according to the following set of rules: 

• Initiate $A_{\mathrm{trip}}(\omega )=1$.

Branching node

• For any branching node at which the trip passes from branch *i* to a different branch *k*, $A_{\mathrm{trip}}(\omega )$ is multiplied by a factor $2p_{k}(\omega )$.

• For any branching node at which the trip approaches a node and reflects off this node back along the same branch *k*, $A_{\mathrm{trip}}(\omega )$ is multiplied by a factor $2p_{k}(\omega )-1$.

Here, the frequency dependent parameter $p_{k}(\omega )$ is defined as

(30) $$p_{k}(\omega )=\frac{z_{k}(\omega )}{\sum_{n}z_{n}(\omega )},\;z_{k}(\omega )=\frac{\gamma_{k}(\omega )}{r_{a,k}},$$

where the sum is over all branches connected to the node.

Terminal

• For every terminal which always reflects any trip, $A_{\mathrm{trip}}$ is multiplied by +1 for the closed-end boundary condition or by −1 for the open-end boundary condition.

Somatic node

• For the somatic node at which the trip passes through the soma from branch *i* to a different branch *k*, $A_{\mathrm{trip}}(\omega )$ is multiplied by a factor $2p_{s,k}(\omega )$.

• For the somatic node at which the trip approaches the soma and reflects off the soma back along the same branch *k*, $A_{\mathrm{trip}}(\omega )$ is multiplied by a factor $2p_{s,k}(\omega )-1$.

Here, the frequency dependent parameter $p_{s,k}(\omega )$ is defined as

(31) $$p_{s,k}(\omega )=\frac{z_{k}(\omega )}{\sum_{n}z_{n}(\omega )+\gamma_{s}(\omega )},\;\gamma_{s}(\omega )=C_{s}\omega +\frac{1}{R_{s}}+\frac{1}{r_{s}+L_{s}\omega },$$

where the sum is over all branches connected to the soma.

GJ node

• For the GJ node at which the trip passes through the gap junction from branch *n* to branch *m*, $A_{\mathrm{trip}}(\omega )$ is multiplied by a factor $p_{\mathrm{GJ},m}(\omega )$. For the GJ node at which the trip passes through the gap junction from branch *m* to branch *n*, $A_{\mathrm{trip}}(\omega )$ is multiplied by a factor $p_{\mathrm{GJ},n}(\omega )$.

• For the GJ node at which the trip approaches the gap junction, passes it and then continues along the same branch *m*, $A_{\mathrm{trip}}(\omega )$ is multiplied by a factor $1-p_{\mathrm{GJ},n}(\omega )$. For the GJ node at which the trip approaches the gap junction, passes it and then continues along the same branch *n*, $A_{\mathrm{trip}}(\omega )$ is multiplied by a factor $1-p_{\mathrm{GJ},m}(\omega )$.

• For the GJ node at which the trip approaches the gap junction and reflects off the gap junction back along the same branch *m*, $A_{\mathrm{trip}}(\omega )$ is multiplied by a factor $-p_{\mathrm{GJ},n}(\omega )$. For the GJ node at which the trip approaches the gap junction and reflects off the gap junction back along the same branch *n*, $A_{\mathrm{trip}}(\omega )$ is multiplied by a factor $-p_{\mathrm{GJ},m}(\omega )$.

Here, parameters $p_{\mathrm{GJ},m}(\omega )$ and $p_{\mathrm{GJ},n}(\omega )$ are defined by Eqs. (26) and (27).

We refer the reader to Coombes et al. [17] for a proof of rules for branching and somatic nodes. In Appendix B, we prove that the rules for generating the trip coefficients at the GJ node satisfy the gap-junctional boundary conditions.

## 4 Application: Two-Cell Network

**Fig. 11:**
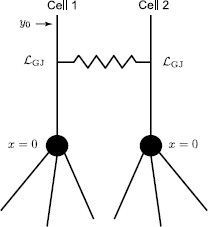
A two-cell model

Here, we demonstrate how the “sum-over-trips” formalism can be applied to a two-cell network for obtaining insight into network response. As an example, we consider a model of two identical cells, each of which consists of a soma and *N* attached semi-infinite dendrites as shown in Fig. 11. The cells are coupled by a dendro-dendritic gap junction located at some distance $L_{\mathrm{GJ}}$ away from their cell bodies. We assume that this network receives an input at the location $y_{0}$. To study the dynamics of this network, we use the “sum-over-trips” framework and construct the Green’s functions ${\hat{G}}_{1}(x,y_{0},\omega )$ and ${\hat{G}}_{2}(x,y_{0},\omega )$ for Cell 1 and Cell 2, respectively. 

### 4.1 Method of Words for Compact Solutions

**Fig. 12:**
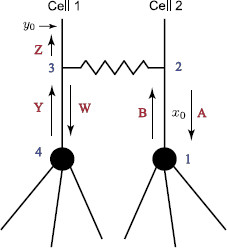
A two-cell model with associated letters

Here, we introduce a method which allows us to construct compact solution forms for the Green’s functions of this two-cell network. We describe this method in detail by constructing the Green’s function ${\hat{G}}_{2}(x_{0},y_{0},\omega )$ for Cell 2 when $x_{0}$ is placed between the soma and the gap-junction as shown in Fig. 12. Introducing points from 1 to 4 on this network, we associate *letters* with different directions as follows: 

• From $x_{0}\to 1$ or from 2→1: letter **A**.

• From $x_{0}\to 2$ or from 1→2: letter **B**.

• From 3→4: letter **W**.

• From 4→3: letter **Y**.

• From $3\to y_{0}$: letter **Z**.

 Then the shortest trip which is a trip from $x_{0}\to 2\to 3\to y_{0}$ is associated with the (ordered) word **BZ** consisting of one syllable. This and any trip associated with a word that starts with the letter **B**, i.e. from $x_{0}\to 2$, and ends with the letter **Z**, i.e. from $3\to y_{0}$, will belong to class 1. Any trip associated with a word which starts from $x_{0}\to 1\to 2$ and ends with $3\to y_{0}$, will belong to class 2. The shortest trip in this class is associated with the word **ABZ**, consisting of two syllables, **AB** and **BZ**. We introduce the following table that associates individual coefficients in the “sum-over-trips” framework with the syllables: 

 As the cells are identical in this network, the parameter $p_{\mathrm{GJ}}(\omega )$ is defined by (18) and

(32) $$p_{s}(\omega )=\frac{\gamma (\omega )/r_{a}}{N\gamma (\omega )/r_{a}+\gamma_{s}(\omega )},$$

where *N* is a number of dendrites attached to each soma and $\gamma_{s}(\omega )$ is given in (31). Then, using the table, it is easy to conclude that for example, the word **BZ** is associated with the coefficient $p_{\mathrm{GJ}}(\omega )$ and the word **ABZ**, consisting of the syllables **AB** and **BZ**, is associated with the coefficient $\left(2p_{s}(\omega )-1)p_{\mathrm{GJ}}(\omega \right)$. We also notice from the table that different coefficients are associated with the syllables **BZ** and **YZ** and, therefore, we need to introduce two additional classes. Class 3 will include the trips with the main skeleton $x_{0}\to 2\to 3\to 4\to 3\to y_{0}$ and the associated word **BWYZ**. Class 4 will include the trips with the main skeleton $x_{0}\to 1\to 2\to 3\to 4\to 3\to y_{0}$ and the associated word **ABWYZ**. Combining the skeleton structures (the shortest words) of the four classes, we have (33)

 Any new word in each class can be formed by adding a combination of syllables **AB** and **WY** into the structure (33). Introducing such additions of *n* combinations of syllables consisting of *k* syllables **AB** and (n−k) syllables **WY** by [⋯⋯] (both syllables **AB** and **WY** can take any position in this sequence of *n* syllables), class 3 and class 4 can be generalised as (34)

 Similarly, we can generalise class 1 and class 2. However, to ensure that the words belong to class 1 and class 2, the syllable **AB** must be at the end of each word in combinations. This can be written as (35)

 where

(36) $${\left[\cdots \;\cdots \right]}^{\prime }=\left[1+[\cdots \;\cdots ]\mathrm{AB}\right].$$

Using combinatorics, we can write

(37) [⋯⋯]=∑k=0n(nk)(AB)k(WY)n−k=(n0)(WY)n+∑k=1n−1(nk)(AB)k(WY)n−k+(nn)(AB)n,

which leads to

(38) [⋯⋯]=(n0)(2ps(ω)−1)n(−pGJ(ω))n−1+∑k=1n−1(nk)(2ps(ω)−1)k(−pGJ(ω))k−1×pGJ(ω)(2ps(ω)−1)n−k(−pGJ(ω))n−k−1+(nn)(2ps(ω)−1)n(−pGJ(ω))n−1=(n0)(2ps(ω)−1)n(−pGJ(ω))n−1−∑k=1n−1(nk)(2ps(ω)−1)n(−pGJ(ω))n−1+(nn)(2ps(ω)−1)n(−pGJ(ω))n−1.

Considering the possible trips in class 3 given by

(39) B[⋯⋯]WYZ

and substituting the expression for [⋯⋯] found in (38), we find

(40) pGJ(ω)(n0)(2ps(ω)−1)n(−pGJ(ω))n−1(−pGJ(ω))(2ps(ω)−1)(1−pGJ(ω))−(−pGJ(ω))∑k=1n−1(nk)(2ps(ω)−1)n(−pGJ(ω))n−1×(−pGJ(ω))(2ps(ω)−1)(1−pGJ(ω))+(−pGJ(ω))(nn)(2ps(ω)−1)n(−pGJ(ω))n−1×pGJ(ω)(2ps(ω)−1)(1−pGJ(ω))=[2n(2ps(ω)−1)n(−pGJ(ω))n]pGJ(ω)(2ps(ω)−1)(1−pGJ(ω)).

Similarly, the possible trips in class 4 given by

(41) AB[⋯⋯]WYZ

generate the trip coefficients

(42) $$\left(2p_{s}(\omega )-1\right)\left[2^{n}{\left(2p_{s}(\omega )-1\right)}^{n}{\left(-p_{\mathrm{GJ}}(\omega )\right)}^{n}\right]p_{\mathrm{GJ}}(\omega )\left(2p_{s}(\omega )-1\right)\left(1-p_{\mathrm{GJ}}(\omega )\right).$$

Expressions (40) and (42) for coefficients in the trips belonging to class 3 and class 4 can now be used in the “sum-over-trips” expansion (29) to obtain

(43) $$\begin{array}{rcl} & & \sum_{n=0}^{\infty }{\left[-2p_{\mathrm{GJ}}(\omega )\left(2p_{s}(\omega )-1\right)\right]}^{n}p_{\mathrm{GJ}}(\omega )\left(2p_{s}(\omega )-1\right)\left(1-p_{\mathrm{GJ}}(\omega )\right)\\ & & \;\times [{\hat{G}}_{\infty }\left(y_{0}-x_{0}+2(n+1)L_{\mathrm{GJ}},\omega \right)\\ & & \;+\left(2p_{s}(\omega )-1\right){\hat{G}}_{\infty }\left(y_{0}+x_{0}+2(n+1)L_{\mathrm{GJ}},\omega \right)], \end{array}$$

where ${\hat{G}}_{\infty }(x,\omega )={\hat{H}}_{\infty }(\gamma (\omega )x)/(D\gamma (\omega ))$ defined by (19). Similarly, we can show that the possible trips in class 1 and class 2 given by

(44) (1+A)B[1+[⋯⋯]AB]Z

generate the terms

(45) $$\begin{array}{rcl} & & p_{\mathrm{GJ}}(\omega ){\hat{G}}_{\infty }(y_{0}-x_{0},\omega )+p_{\mathrm{GJ}}(\omega )\left(2p_{s}(\omega )-1\right){\hat{G}}_{\infty }(y_{0}+x_{0},\omega )\\ & & \;+\sum_{n=0}^{\infty }{\left[-2p_{\mathrm{GJ}}(\omega )\left(2p_{s}(\omega )-1\right)\right]}^{n}p_{\mathrm{GJ}}(\omega )\left(-p_{\mathrm{GJ}}(\omega )\right)\left(2p_{s}(\omega )-1\right)\\ & & \;\times [{\hat{G}}_{\infty }\left(y_{0}-x_{0}+2(n+1)L_{\mathrm{GJ}},\omega \right)\\ & & \;+\left(2p_{s}(\omega )-1\right){\hat{G}}_{\infty }\left(y_{0}+x_{0}+2(n+1)L_{\mathrm{GJ}},\omega \right)]. \end{array}$$

Combining together (43) and (45), we obtain

(46) $$\begin{array}{rcl} {\hat{G}}_{2}(x_{0},y_{0},\omega ) & = & p_{\mathrm{GJ}}(\omega ){\hat{G}}_{\infty }(y_{0}-x_{0},\omega )+p_{\mathrm{GJ}}(\omega )\left(2p_{s}(\omega )-1\right){\hat{G}}_{\infty }(y_{0}+x_{0},\omega )\\ & & +\sum_{n=0}^{\infty }{\left[-2p_{\mathrm{GJ}}(\omega )\left(2p_{s}(\omega )-1\right)\right]}^{n}p_{\mathrm{GJ}}(\omega )\left(2p_{s}(\omega )-1\right)\\ & & \times \{\left(-p_{\mathrm{GJ}}(\omega )\right)[{\hat{G}}_{\infty }\left(y_{0}-x_{0}+2(n+1)L_{\mathrm{GJ}},\omega \right)\\ & & +\left(2p_{s}(\omega )-1\right){\hat{G}}_{\infty }\left(y_{0}+x_{0}+2(n+1)L_{\mathrm{GJ}},\omega \right)]\\ & & +\left(1-p_{\mathrm{GJ}}(\omega )\right)[{\hat{G}}_{\infty }\left(y_{0}-x_{0}+2(n+1)L_{\mathrm{GJ}},\omega \right)\\ & & +\left(2p_{s}(\omega )-1\right){\hat{G}}_{\infty }\left(y_{0}+x_{0}+2(n+1)L_{\mathrm{GJ}},\omega \right)]\}. \end{array}$$

Terms in {⋯} in (46) represent multiple trips in each of four classes and since there is a match in the length of trips among different classes, Eq. (46) can be simplified as

(47) $$\begin{array}{rcl} {\hat{G}}_{2}(x_{0},y_{0},\omega ) & = & p_{\mathrm{GJ}}(\omega )\left[{\hat{G}}_{\infty }(y_{0}-x_{0},\omega )+\left(2p_{s}(\omega )-1\right){\hat{G}}_{\infty }(y_{0}+x_{0},\omega )\right]\\ & & +\sum_{n=0}^{\infty }2^{n}{\left(-p_{\mathrm{GJ}}(\omega )\left(2p_{s}(\omega )-1\right)\right)}^{n+1}\left(2p_{\mathrm{GJ}}(\omega )-1\right)\\ & & \times [{\hat{G}}_{\infty }\left(y_{0}-x_{0}+2(n+1)L_{\mathrm{GJ}},\omega \right)\\ & & +\left(2p_{s}(\omega )-1\right){\hat{G}}_{\infty }\left(y_{0}+x_{0}+2(n+1)L_{\mathrm{GJ}},\omega \right)]. \end{array}$$

Using this method of ‘words’, we can construct compact solution forms for the Green’s function for each of these two cells for any combinations of input, *y*, and output, *x*, locations. For example, placing $x_{0}$ in Cell 1 between its soma and the gap-junction we obtain

(48) $$\begin{array}{rcl} {\hat{G}}_{1}(x_{0},y_{0},\omega ) & = & \left(1-p_{\mathrm{GJ}}(\omega )\right)\left[{\hat{G}}_{\infty }(y_{0}-x_{0},\omega )+\left(2p_{s}(\omega )-1\right){\hat{G}}_{\infty }(y_{0}+x_{0},\omega )\right]\\ & & +\sum_{n=0}^{\infty }2^{n}{\left(-p_{\mathrm{GJ}}(\omega )\left(2p_{s}(\omega )-1\right)\right)}^{n+1}\left(1-2p_{\mathrm{GJ}}(\omega )\right)\\ & & \times [{\hat{G}}_{\infty }\left(y_{0}-x_{0}+2(n+1)L_{\mathrm{GJ}},\omega \right)\\ & & +\left(2p_{s}(\omega )-1\right){\hat{G}}_{\infty }\left(y_{0}+x_{0}+2(n+1)L_{\mathrm{GJ}},\omega \right)]. \end{array}$$

### 4.2 Network Dynamics

To study the role of a gap-junction in this two-cell network model, we focus on the Green’s functions at the somas of these two cells in response to a stimulus at the location $y_{0}$. Using Eqs. (47) and (48), we obtain the following somatic response functions:

(49) $$\begin{array}{rcl} {\hat{G}}_{1}(0,y_{0},\omega ) & = & 2p_{s}(\omega )\left(1-p_{\mathrm{GJ}}(\omega )\right){\hat{G}}_{\infty }(y_{0},\omega )\\ & & +\sum_{n=0}^{\infty }2^{n}{\left(-p_{\mathrm{GJ}}(\omega )\left(2p_{s}(\omega )-1\right)\right)}^{n+1}\left(1-2p_{\mathrm{GJ}}(\omega )\right)\\ & & \times 2p_{s}(\omega ){\hat{G}}_{\infty }\left(y_{0}+2(n+1)L_{\mathrm{GJ}},\omega \right), \end{array}$$

and

(50) $$\begin{array}{rcl} {\hat{G}}_{2}(0,y_{0},\omega ) & = & 2p_{s}(\omega )p_{\mathrm{GJ}}(\omega ){\hat{G}}_{\infty }(y_{0},\omega )\\ & & +\sum_{n=0}^{\infty }2^{n}{\left(-p_{\mathrm{GJ}}(\omega )\left(2p_{s}(\omega )-1\right)\right)}^{n+1}\left(2p_{\mathrm{GJ}}(\omega )-1\right)\\ & & \times 2p_{s}(\omega ){\hat{G}}_{\infty }\left(y_{0}+2(n+1)L_{\mathrm{GJ}},\omega \right). \end{array}$$

Resonant properties of each cell are analysed by studying a preferred frequency $\Omega_{0}$ for each cell. This is defined as the frequency at which the corresponding power function, $P_{1}(\omega )={\left|{\hat{G}}_{1}(0,y_{0},\omega )\right|}^{2}$ for Cell 1 and $P_{2}(\omega )={\left|{\hat{G}}_{2}(0,y_{0},\omega )\right|}^{2}$ for Cell 2, reaches its maximum. This means that $\Omega_{0}$ for each soma is simply a solution of one of the corresponding equations, $\partial P_{1}(\omega )/\partial \omega =0$ and $\partial P_{2}(\omega )/\partial \omega =0$.

**Fig. 13:**
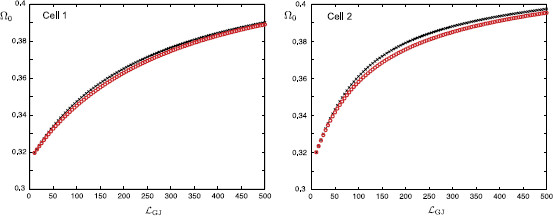
Values of $\Omega_{0}$ at the soma of each cell as a function of $L_{\mathrm{GJ}}$ when $y_{0}=L_{\mathrm{GJ}}+10\text{ μm}$ and $R_{\mathrm{GJ}}=100\text{ M}\Omega$ (*red circles*), $R_{\mathrm{GJ}}=1000\text{ M}\Omega$ (*black crosses*). Passive somas with parameters: diameter $a_{s}=25\text{ μm}$, $C_{s}=1{\text{ μF/cm}}^{2}$, $R_{s}=2000\;\Omega {\text{ cm}}^{2}$. Resonant dendrites (N=4) with parameters given in Fig. 4

**Fig. 14:**
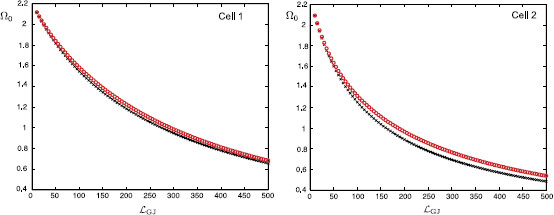
Values of $\Omega_{0}$ at the soma of each cell as a function of $L_{\mathrm{GJ}}$ when $y_{0}=L_{\mathrm{GJ}}+10\text{ μm}$ and $R_{\mathrm{GJ}}=100\text{ M}\Omega$ (*red circles*), $R_{\mathrm{GJ}}=1000\text{ M}\Omega$ (*black crosses*). Resonant somas with parameters: diameter $a_{s}=25\text{ μm}$, $C_{s}=1{\text{ μF/cm}}^{2}$, $R_{s}=2000\;\Omega {\text{ cm}}^{2}$, $r_{s}=1\;\Omega {\text{ cm}}^{2}$, $L_{s}=0.1{\text{ H cm}}^{2}$. Passive dendrites (N=4) with parameters given in Fig. 4

**Fig. 15:**
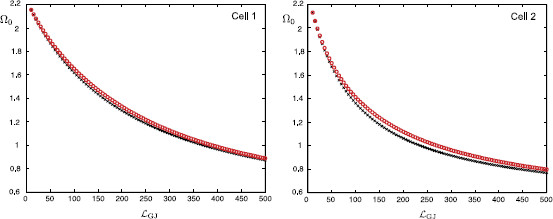
Values of $\Omega_{0}$ at the soma of each cell as a function of $L_{\mathrm{GJ}}$ when $y_{0}=L_{\mathrm{GJ}}+10\text{ μm}$ and $R_{\mathrm{GJ}}=100\text{ M}\Omega$ (*red circles*), $R_{\mathrm{GJ}}=1000\text{ M}\Omega$ (*black crosses*). Resonant somas with parameters: diameter $a_{s}=25\text{ μm}$, $C_{s}=1{\text{ μF/cm}}^{2}$, $R_{s}=2000\;\Omega {\text{ cm}}^{2}$, $r_{s}=1\;\Omega {\text{ cm}}^{2}$, $L_{s}=0.1{\text{ H cm}}^{2}$. Resonant dendrites (N=4) with parameters given in Fig. 4

**Fig. 16:**
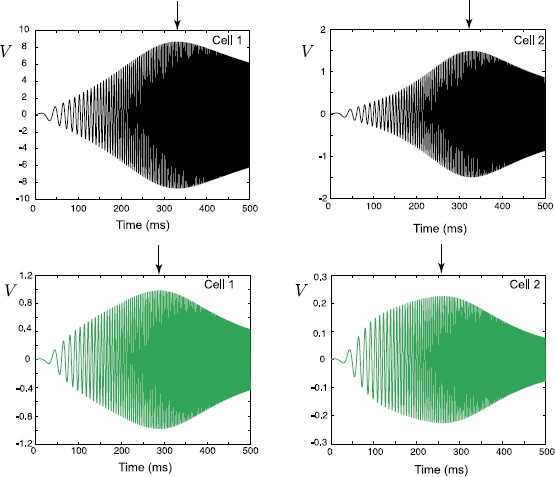
Voltage profiles in the somas of cells in response to a stimulus $I_{\mathrm{chirp}}(t)$ applied at the location $y_{0}=L_{\mathrm{GJ}}+10\text{ μm}$. Cells’ parameters as in Fig. 15, $R_{\mathrm{GJ}}=100\text{ M}\Omega$, $\omega_{\mathrm{chirp}}=0.003$, $A_{\mathrm{chirp}}=1\text{ nA}$. *Black curves*: $L_{\mathrm{GJ}}=50\text{ μm}$, *green curves*: $L_{\mathrm{GJ}}=500\text{ μm}$

In Fig. 13, we plot how this preferred frequency $\Omega_{0}$ varies as a function of the distance of the gap-junction away from each soma. These plots are obtained for the case of a passive soma and resonant dendrites. In Fig. 14 we demonstrate how $\Omega_{0}$ is affected if a resonant soma and passive dendrites are considered. Finally, in Fig. 15, we plot $\Omega_{0}(L_{\mathrm{GJ}})$ when each soma and dendritic branch is modelled with resonant membrane. All these three figures clearly demonstrate that the somatic response in each cell strongly depends on the location of the gap-junction and it is tuned to be maximised for different frequencies. This can be shown by applying a chirp stimulus at the location $y_{0}$ and plotting the somatic voltage for each cell (see Fig. 16). Resonances in each cell occur at different times as predicted by Fig. 15. All these figures are constructed for truncated series solutions (49), (50) when the index *n* increases up to 20, although it is possible to show that the solutions rapidly converge for a much smaller *n* such as n=10. 

## 5 Discussion

In this paper, we have generalised the “sum-over-trips” formalism for single dendritic trees to cover networks of gap-junction coupled resonant neurons. With the use of ideas from combinatorics, we have also introduced a so-called method of ‘words’ that allows for a compact representation of the Green’s function network response formulas. This has allowed us to determine that the position of a dendro-dendritic gap junction can be used to tune the preferred frequency at the cell body. Moreover we have been able to generate mathematical formula for this dependence without recourse to direct numerical simulations of the physical model. One clear prediction is that the preferred frequency increases with distance of the gap junction from the soma in a model with passive soma and resonant dendrites. In contrast for a system with a resonant soma and passive or resonant dendrite, the preferred frequency decreases as the gap junction is placed further away from the cell body.

There are a number of natural extensions of the work in this paper. One is an application to more realistic network geometries or more than just two neurons, as may be found in retinal networks. Here, it would also be interesting to exploit any network symmetries (either arising from the identical nature of the cells, their shapes, or the topology of their coupling) to allow for the compact representation of network response (and further utilising the method of ‘words’). Another is to incorporate a model of an active soma whilst preserving some measure of analytical tractability. Schwemmer and Lewis [21] have recently achieved this for a single unbranched cable model by coupling it to an integrate-and-fire soma model. The merger of our approach with theirs may pave the way for understanding *spiking* networks of gap junction coupled dendritic trees. Moreover, by using the techniques developed by them in [22] (using weakly coupled oscillator theory) we may further shed light on the role of dendro-dendritic coupling in contributing to the robustness of phase-locking in oscillatory networks. 

## Appendix A: Two Simplified Identical Cells with Passive Membrane

Equations (14)–(16) with $\gamma^{2}(\omega )=(1/\tau +\omega )/D$ provide the solutions of a model in Fig. 2 with passive membrane. We introduce the function

(51) $$\hat{F}(x,\omega ,q)=\frac{1}{\gamma (\omega )+q}\frac{e^{-\gamma (\omega )|x|}}{2D\gamma (\omega )},$$

and its inverse Laplace transform

(52) $$\begin{array}{rcl} F(x,t,q) & = & L^{-1}\left[\hat{F}(x,\omega ,q)\right]\\ & = & \frac{1}{2}e^{|x|q}e^{(q^{2}D-1/\tau )t}\mathrm{erfc}\left(q\sqrt{D}+\frac{\left|x\right|}{2\sqrt{Dt}}\right)\Theta (t). \end{array}$$

Then the Green’s function on each segment can be found in closed form as

(53) $$\begin{array}{rcl} G_{m^{-}}(x,y,t) & = & L^{-1}\left[{\hat{G}}_{m^{-}}(x,y,\omega )\right]\\ & = & \frac{1}{C}\left[G_{\infty }(x-y,t)-\frac{r_{a}}{2R_{\mathrm{GJ}}}F(x+y,t,r_{a}/R)\right], \end{array}$$

(54) $$\begin{array}{rcl} G_{m^{+}}(x,y,t) & = & L^{-1}\left[{\hat{G}}_{m^{+}}(x,y,\omega )\right]\\ & = & \frac{1}{C}\left[G_{\infty }(x+y,t)-\frac{r_{a}}{2R_{\mathrm{GJ}}}F(x+y,t,r_{a}/R)\right], \end{array}$$

(55) $$\begin{array}{rcl} G_{n^{\pm }}(x,y,t) & = & L^{-1}\left[{\hat{G}}_{n^{\pm }}(x,y,\omega )\right]\\ & = & \frac{1}{C}\left[\frac{r_{a}}{2R_{\mathrm{GJ}}}F(x+y,t,r_{a}/R)\right], \end{array}$$

where $G_{\infty }(x,t)$ is the Green’s function of the passive infinite dendritic cable,

(56) $$G_{\infty }(x,t)=L^{-1}\left[\frac{e^{-\gamma (\omega )|x|}}{2D\gamma (\omega )}\right]=\frac{1}{\sqrt{4\pi Dt}}e^{-t/\tau }e^{-x^{2}/(4Dt)}\Theta (t).$$

If an external stimulus $I_{\mathrm{inj},m^{-}}(x,t)=\delta (x-y)I(t)$ has a form of a rectangular pulse with $I(t)=\eta_{0}\Theta (t)\Theta (\tau_{R}-t)$, the voltage response on each segment can also be found in closed form:

(57) $$\begin{array}{rcl} V_{m^{-}}(x,t) & = & \left[B(x-y,t)-B(x-y,t-\tau_{R}\right)\\ & & -\left(P(x+y,t)-P(x+y,t-\tau_{R})\right)]/C, \end{array}$$

(58) $$\begin{array}{rcl} V_{m^{+}}(x,t) & = & \left[B(x+y,t)-B(x+y,t-\tau_{R}\right)\\ & & -\left(P(x+y,t)-P(x+y,t-\tau_{R})\right)]/C, \end{array}$$

(59) $$V_{n^{\pm }}(x,t)=\left[P(x+y,t)-P(x+y,t-\tau_{R})\right]/C,$$

where

(60) $$\begin{array}{rcl} B(x,t) & = & \frac{\eta_{0}}{4\sqrt{D/\tau }}[e^{-|x|/\sqrt{D\tau }}\mathrm{erfc}\left(\frac{\left|x\right|}{2\sqrt{Dt}}-\sqrt{t/\tau }\right)\\ & & -e^{|x|/\sqrt{D\tau }}\mathrm{erfc}\left(\frac{\left|x\right|}{2\sqrt{Dt}}+\sqrt{t/\tau }\right)]\Theta (t), \end{array}$$

(61) $$P(x,t)=\frac{\eta_{0}r_{a}}{2DR_{\mathrm{GJ}}}\left[aF(x,t,r_{a}/R_{\mathrm{GJ}})+bF(x,t,\sqrt{\varepsilon })+cF(x,t,-\sqrt{\varepsilon })\right],$$

and

(62) $$\begin{array}{rl} \varepsilon & =\frac{1}{D\tau },\;a=\frac{1}{{\left(r_{a}/R_{\mathrm{GJ}}\right)}^{2}-\varepsilon },\\ b & =\frac{1}{2\sqrt{\varepsilon }(\sqrt{\varepsilon }-r_{a}/R_{\mathrm{GJ}})},\;c=\frac{1}{2\sqrt{\varepsilon }(\sqrt{\varepsilon }+r_{a}/R_{\mathrm{GJ}})}. \end{array}$$

These solutions generalise earlier results of Harris and Timofeeva [23] applicable to a neural network, but with gap-junctional coupling at tip-to-tip contacts of two branches. 

## Appendix B: Proof of the “Sum-over-Trips” Rules at the Gap Junction

As the Green’s function is constructed in the Laplace domain, the rules at the gap-junction need to satisfy the following boundary conditions (after a length of each branch, say labelled by *k*, of the tree is re-scaled as $X=\gamma_{k}(\omega )x$, $x\in [0,L_{k}]$):

(63) $$G_{m^{-}j}(0,Y,\omega )=G_{m^{+}j}(0,Y,\omega ),$$

(64) $$G_{n^{-}j}(0,Y,\omega )=G_{n^{+}j}(0,Y,\omega ),$$

and

(65) $$\begin{array}{rcl} & & \frac{\gamma_{m}(\omega )}{r_{a,m}}\left({\frac{\partial G_{m^{-}j}}{\partial X}|}_{X=0}+{\frac{\partial G_{m^{+}j}}{\partial X}|}_{X=0}\right)\\ & & \;=g_{\mathrm{GJ}}\left(G_{m^{-}j}(0,Y,\omega )-G_{n^{-}j}(0,\omega )\right), \end{array}$$

(66) $$\begin{array}{rcl} & & \frac{\gamma_{n}(\omega )}{r_{a,n}}\left({\frac{\partial G_{n^{-}j}}{\partial X}|}_{X=0}+{\frac{\partial G_{n^{+}j}}{\partial X}|}_{X=0}\right)\\ & & \;=g_{\mathrm{GJ}}\left(G_{n^{-}j}(0,Y,\omega )-G_{m^{-}j}(0,Y,\omega )\right). \end{array}$$

We prove here that the rules for generating the trip coefficients are consistent with these boundary conditions.

**Fig. 17:**
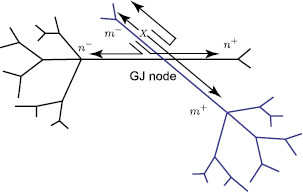
*The GJ node* with possible trips in its proximity

Let *X* denote the distance away from the GJ node along the segment $m^{-}$ (see Fig. 17). The location of the stimulus $Y=\gamma_{j}(\omega )y$, the segment number *j* and the variable *ω* are all considered to be arbitrary. Suppose that we sum all the trips starting from the GJ node itself and ending at point *Y* on branch *j*. We denote the result of summing over all trips that initially leave the GJ node along segment $m^{-}$ by $Q_{m^{-}j}(0,Y,\omega )$, along segment $m^{+}$ by $Q_{m^{+}j}(0,Y,\omega )$, along segment $n^{-}$ by $Q_{n^{-}j}(0,Y,\omega )$ and along segment $n^{+}$ by $Q_{n^{+}j}(0,Y,\omega )$. 

Trips that start out from *X* and move away from the GJ node are identical to trips that start out from the GJ node itself along segment $m^{-}$. The only difference is that the trips in the first case are shorter by the length *X*. We denote the sum of such shortened trips by $Q_{m^{-}j}(-X,Y,\omega )$. The argument −*X* means that a distance *X* has to be subtracted from the length of each trip summed to compute  (and not that the trips start at the point −*X*).

Trips that start out from *X* by moving toward the GJ node and then reflecting back along segment $m^{-}$ are also identical to trips that start out from the GJ node along segment $m^{-}$ except that these are longer by the length *X*. In addition, because of the reflection from the GJ node these trips pick up a factor $-p_{\mathrm{GJ},n}(\omega )$ according to the “sum-over-trips” rules. Therefore, the contribution to the solution $G_{m^{-}j}(X,Y,\omega )$ from those trips is $-p_{\mathrm{GJ},n}(\omega )Q_{m^{-}j}(X,Y,\omega )$. Trips that start out from *X* by moving toward the GJ node and then continue moving along branch *m*, i.e. on segment $m^{+}$, pick up a factor $1-p_{\mathrm{GJ},n}(\omega )$ and the sum of such trips is given by $\left(1-p_{\mathrm{GJ},n}(\omega ))Q_{m^{+}j}(X,Y,\omega \right)$. Finally, trips that start from *X*, move toward the GJ node and then leave the GJ node by moving out along segment $n^{-}$ or $n^{+}$ pick up a factor $p_{\mathrm{GJ},n}(\omega )$ and contribute to the solution $G_{m^{-}j}(X,Y,\omega )$ by the terms $p_{\mathrm{GJ},n}(\omega )Q_{n^{-}j}(X,Y,\omega )$ or $p_{\mathrm{GJ},n}(\omega )Q_{n^{+}j}(X,Y,\omega )$.

The full solution $G_{m^{-}j}(X,Y,\omega )$ includes the contributions from all different types of trips we have been discussing. Thus,

(67) $$\begin{array}{rcl} G_{m^{-}j}(X,Y,\omega ) & = & \frac{1}{D_{j}\gamma_{j}(\omega )}[Q_{m^{-}j}(-X,Y,\omega )+\left(-p_{\mathrm{GJ},n}(\omega )\right)Q_{m^{-}j}(X,Y,\omega )\\ & & +\left(1-p_{\mathrm{GJ},n}(\omega )\right)Q_{m^{+}j}(X,Y,\omega )+p_{\mathrm{GJ},n}(\omega )Q_{n^{-}j}(X,Y,\omega )\\ & & +p_{\mathrm{GJ},n}(\omega )Q_{n^{+}j}(X,Y,\omega )]. \end{array}$$

The functions  in this formula consist of infinite sums over trips, but we do not need to know what they are to show that the solution $G_{m^{-}j}(X,Y,\omega )$ satisfies the GJ node boundary conditions. At the GJ node, we have

(68) $$\begin{array}{rcl} G_{m^{-}j}(0,Y,\omega ) & = & \frac{1}{D_{j}\gamma_{j}(\omega )}[\left(1-p_{\mathrm{GJ},n}(\omega )\right)Q_{m^{-}j}(0,Y,\omega )\\ & & +\left(1-p_{\mathrm{GJ},n}(\omega )\right)Q_{m^{+}j}(0,Y,\omega )\\ & & +p_{\mathrm{GJ},n}(\omega )\left(Q_{n^{-}j}(0,Y,\omega )+Q_{n^{+}j}(0,Y,\omega )\right)]. \end{array}$$

Considering the solution $G_{m^{+}j}(0,Y,\omega )$ instead gives us the same expression as in (68) and, therefore, $G_{m^{-}j}(X,Y,\omega )$ obeys the boundary condition (63). Similarly, we can show that

(69) $$\begin{array}{rcl} G_{n^{-}j}(0,Y,\omega ) & = & G_{n^{+}j}(0,Y,\omega )\\ & = & \frac{1}{D_{j}\gamma_{j}(\omega )}[\left(1-p_{\mathrm{GJ},m}(\omega )\right)Q_{n^{-}j}(0,Y,\omega )\\ & & +\left(1-p_{\mathrm{GJ},m}(\omega )\right)Q_{n^{+}j}(0,Y,\omega )\\ & & +p_{\mathrm{GJ},m}(\omega )\left(Q_{m^{-}j}(0,Y,\omega )+Q_{m^{+}j}(0,Y,\omega )\right)], \end{array}$$

which satisfies the boundary condition (64).

To prove the boundary condition (65) we use Eq. (67) to find that

(70) $$\begin{array}{rcl} {\frac{\partial G_{m^{-}j}}{\partial X}|}_{X=0} & = & \frac{1}{D_{j}\gamma_{j}(\omega )}[{\frac{\partial Q_{m^{-}j}(-X,Y,\omega )}{\partial X}|}_{X=0}\\ & & -p_{\mathrm{GJ},n}(\omega ){\frac{\partial Q_{m^{-}j}(X,Y,\omega )}{\partial X}|}_{X=0}\\ & & +\left(1-p_{\mathrm{GJ},n}(\omega )\right){\frac{\partial Q_{m^{+}j}(X,Y,\omega )}{\partial X}|}_{X=0}\\ & & +p_{\mathrm{GJ},n}(\omega ){\left(\frac{\partial Q_{n^{-}j}(X,Y,\omega )}{\partial X}\right|}_{X=0}\\ & & +{\frac{\partial Q_{n^{+}j}(X,Y,\omega )}{\partial X}|}_{X=0})]. \end{array}$$

Using the following properties for the term $Q_{kj}(X,Y,\omega )$, $k\in \{m^{-},m^{+},n^{-},n^{+}\}$,

(71) $$\frac{\partial Q_{kj}(X,Y,\omega )}{\partial X}=-Q_{kj}(X,Y,\omega ),$$

(72) $$\frac{\partial Q_{kj}(-X,Y,\omega )}{\partial X}=Q_{kj}(X,Y,\omega ),$$

Eq. (70) can be simplified as

(73) $$\begin{array}{rcl} {\frac{\partial G_{m^{-}j}}{\partial X}|}_{X=0} & = & \left(1+p_{\mathrm{GJ},n}(\omega )\right)Q_{m^{-}}(0,Y,\omega )-\left(1-p_{\mathrm{GJ},n}(\omega )\right)Q_{m^{+}}(0,Y,\omega )\\ & & -p_{\mathrm{GJ},n}(\omega )\left(Q_{n^{-}}(0,Y,\omega )+Q_{n^{+}}(0,Y,\omega )\right). \end{array}$$

Similarly,

(74) $$\begin{array}{rcl} {\frac{\partial G_{m^{+}j}}{\partial X}|}_{X=0} & = & \left(1+p_{\mathrm{GJ},n}(\omega )\right)Q_{m^{+}}(0,Y,\omega )-\left(1-p_{\mathrm{GJ},n}(\omega )\right)Q_{m^{-}}(0,Y,\omega )\\ & & -p_{\mathrm{GJ},n}(\omega )\left(Q_{n^{-}}(0,Y,\omega )+Q_{n^{+}}(0,Y,\omega )\right). \end{array}$$

Substituting (73) and (74) together with (68) and (69) in Eq. (65) gives us the right equality. Similarly, we can prove the boundary condition (66).

## Competing Interests

The authors declare that they have no competing interests.

## Authors’ Contributions

YT, SC and DM contributed equally. All authors read and approved the final manuscript.
